# Severe Illness Associated with Synthetic Cannabinoid Use — Brunswick, Georgia, 2013

**Published:** 2013-11-22

**Authors:** Cherie Drenzek, Robert J. Geller, Alaina Steck, Justin Arnold, Gaylord Lopez, Roy Gerona, Laura Edison

**Affiliations:** Georgia Dept of Public Health; Georgia Poison Center; Dept of Laboratory Medicine, Univ of California, San Francisco; EIS Officer, CDC

On August 23, 2013, the Georgia Poison Center was notified of eight persons examined in an emergency department in Brunswick, Georgia, after smoking or inhaling fumes from synthetic cannabinoids. The Georgia Poison Center notified the Georgia Drug and Narcotics Agency, which informed the Georgia Department of Public Health (DPH). The Brunswick emergency department was asked to report any additional patients who reported use of synthetic cannabinoid to the Coastal District Health Department. DPH investigators reviewed recent medical records of patients who had gone to the emergency department and found that 22 patients had been examined after using synthetic cannabinoids during August 22–September 9, 2013.

The 22 patients were aged 16–57 years (median: 25 years); 18 (82%) were male. Patients experienced hyperglycemia (13 [59%]), hypokalemia (nine [41%]), acidosis (seven [32%]), tachycardia (13 [59%]), nausea/vomiting (eight [36%]), confusion/disorientation (seven [32%]), aggression (seven [32%]), somnolence/unresponsiveness (seven [32%]), and seizures (three [14%]). Complications included pneumonia (two patients), rhabdomyolysis (one), and myocardial infarction (one). Six (27%) patients were admitted to the intensive care unit; five (23%) required assisted ventilation; none died. Serum from seven of the initial eight patients was tested for synthetic cannabinoid by the Clinical and Environmental Toxicology Laboratory at the University of California, San Francisco. Five tested positive for ADB-PINACA (N-(1-amino-3,3-dimethy-1-oxobutan-2-yl)-1-pentyl-1H-indazole-3-carboxamide), a previously unrecognized synthetic cannabinoid related to indole compounds recently identified in Europe and Japan (1).

Law enforcement authorities removed the synthetic cannabinoid from the implicated Brunswick smoke shop,* which sold all types of tobacco products and smoking paraphernalia. The product, “Crazy Clown,” was tested by the Georgia Bureau of Investigation Crime Laboratory, which identified ADB-PINACA, an indazole classified under Georgia law as Schedule 1 on the basis of its close relationship to Schedule 1 compounds already specified. The smoke shop owners were charged on September 10, 2013, with possession of a Schedule 1 controlled substance with intent to distribute; no additional patients who used synthetic cannabinoids have been reported by the ED.

Synthetic cannabinoids are designer drugs often smoked as a marijuana alternative. Despite laws prohibiting synthetic cannabinoid sales, they are still widely available, and recent increases in reports of synthetic cannabinoid use and adverse health effects have occurred (2,3). Common adverse effects include altered mental status and tachycardia. Clinicians examining patients with suspected drug abuse and these symptoms should consider synthetic cannabinoid intoxication (4). Public health authorities can raise awareness of adverse events associated with synthetic cannabinoids and establish mechanisms for surveillance by partnering with poison centers, health-care providers, and law enforcement.

## Footnotes

* The initial eight patients reported buying the synthetic cannabinoid from the same smoke shop. Additional patients interviewed also named the smoke shop as the source.
